# Electroosmotic Flow of Viscoelastic Fluid in a Nanoslit

**DOI:** 10.3390/mi9040155

**Published:** 2018-03-29

**Authors:** Lanju Mei, Hongna Zhang, Hongxia Meng, Shizhi Qian

**Affiliations:** 1 Department of Mechanical and Aerospace Engineering, Old Dominion University, Norfolk, VA 23529, USA; lmei004@odu.edu; 2 Sino-French Institute of Nuclear Engineering and Technology, Sun Yat-sen University, Zhuhai 519082, China; zhanghn26@mail.sysu.edu.cn; 3 School of Power and Mechanical Engineering, Wuhan University, Wuhan 430072, China; mhx2005@whu.edu.cn

**Keywords:** electroosmotic flow, viscoelastic fluid, nanoslit, Linear Phan-Thien-Tanner (LPTT) model, electrical double layer

## Abstract

The electroosmotic flow (EOF) of viscoelastic fluid in a long nanoslit is numerically studied to investigate the rheological property effect of Linear Phan-Thien-Tanner (LPTT) fluid on the fully developed EOF. The non-linear Poisson-Nernst-Planck equations governing the electric potential and the ionic concentration distribution within the channel are adopted to take into account the effect of the electrical double layer (EDL), including the EDL overlap. When the EDL is not overlapped, the velocity profiles for both Newtonian and viscoelastic fluids are plug-like and increase sharply near the charged wall. The velocity profile resembles that of pressure-driven flow when the EDL is overlapped. Regardless of the EDL thickness, apparent increase of velocity is obtained for viscoelastic fluid of larger Weissenberg number compared to the Newtonian fluid, indicating the shear thinning behavior of the LPTT fluid. The effect of the Weissenberg number on the velocity distribution is less significant as the degree of EDL overlapping increases, due to the overall decrease of the shear rate. The increase (decrease) of polymer extensibility (viscosity ratio) also enhances the EOF of viscoelastic fluid.

## 1. Introduction

Over recent decades, nanofluidics has undergone significant development due to the advances in nanofabrication and its promising applications in (bio)nanoparticle sensing and detection [1,2,3], manipulation of charged analytes [4,5], sequencing of single DNA molecules [6,7], etc. Particularly, electrokinetic transport of ions and fluid in nanofluidic devices is of fundamental and practical importance [8,9,10]. This phenomenon was first reported by Reuss [11], and later has been extensively studied both experimentally [12,13,14] and theoretically [15,16,17,18,19,20]. As the characteristic length scale of the nanofluidic devices is on the nanoscale, the thickness of the electrical double layer (EDL), comprising of an immobile Stern layer and diffusive layer, becomes comparable to the characteristic size of nanochannel or nanopore, especially at relatively low bulk concentration, resulting in the EDL overlapping [20,21,22].

Most of the theoretical studies on electroosmotic flow (EOF) in the literature assume that the fluid follows the Newtonian model. However, in real nanofluidic applications, complex solutions such as polymer and DNA solutions are frequently involved, and they impart distinctively different characterization from the Newtonian fluid [23,24], including shear-rate-dependent viscosity, memory effects, normal stress difference, etc. Thus, the EOF of these solutions will be different from that of Newtonian fluid. Recently, some theoretical studies on EOF in microfluidics taking into account of the non-Newtonian effect have emerged. For example, Das et al. [25] derived the analytical solution of EOF of non-Newtonian fluid in a rectangular microchannel using the power-law model. Zimmerman et al. [26] conducted finite element simulation of electrokinetic flow of Carreau-type fluid in a microchannel with T-junction, which contributed to the design of highly efficient viscometric devices. Olivares et al. [27] explained the modeling of the EOF using polymer adsorption and depletion in the EDL. Zhao et al. [28] analyzed the EOF of power-law fluids in a slit microchannel and examined the effects of flow behavior index, double layer thickness and electric field. Zhao et al. [24] numerically investigated the electro-osmotic mobility with a more general Carreau non-Newtonian model. The aforementioned studies are limited to the simple non-Newtonian fluid model that does not exhibit elastic characteristics. For pure EOF of Phan-Thien-Tanner (PTT) fluid, Park et al. [29] firstly derived the Helmholtz-Smoluchowski velocity and provided a simple method to calculate the volumetric flow rate in microchannel. For viscoelastic fluid with mixed pressure-electroosmotic driving force, Park et al. [30] also investigated viscoelastic EOF in a microchannel. Afonso et al. [31,32] reported analytical solutions for EOF of viscoelastic fluid in a microchannel and between two concentric cylinders using the PTT model and Finitely Extensible Nonlinear Elastic with Peterlin closure (FENE-P) model. Based on the earlier work, Afonso et al. [33] also investigated the EOF of viscoelastic fluid in a microchannel with asymmetric zeta potential, and Sousa et al. [34] derived an analytical solution taking into account the wall depleted layer. Dhinakaran et al. [35] extended the work of Afonso et al. [31] and analytically analyzed the steady EOF of viscoelastic fluid in a microchannel with the PTT model by taking into account the full Gordon-Schowalter convective derivative. 

Most of the studies on EOF of viscoelastic fluid in a microchannel assume relatively small surface potential and thin EDL so that the Poisson-Nernst-Planck equations can be simplified. The condition with EDL thickness comparable to the channel height, which is typical in modern nanofluidics [4,13,36,37], has not been studied. In this study, we numerically study the EOF of viscoelastic fluid with the PTT constitutive model in a nanoslit under different EDL conditions. The effects of EDL thickness, Weissenberg number (*Wi*), viscosity ratio and polymer extensibility parameter on EOF velocity profile and dynamic viscosity are examined. The rest of this paper is organized as follows. The problem under consideration is physically described and the governing equations are presented in Section 2. Then, the accuracy of the numerical method is verified in Section 3. Finally, the parametric study results and the conclusions are presented.

## 2. Mathematical Model

**Figure 1:**
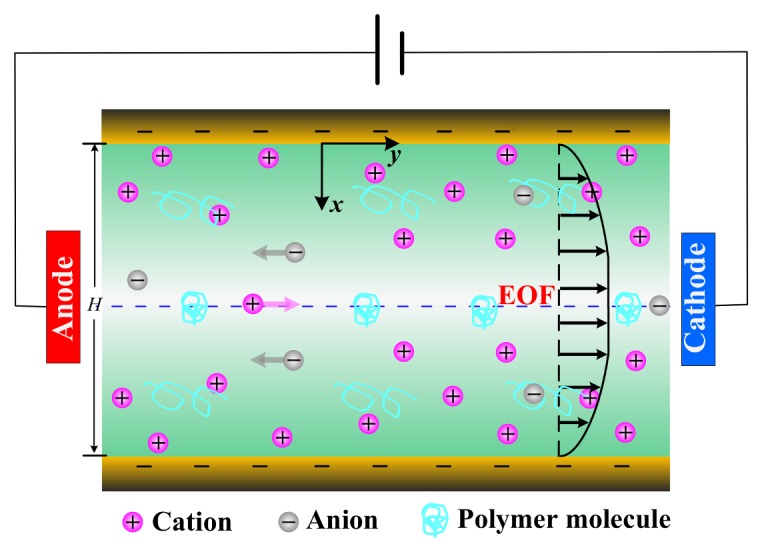
Schematic diagram of EOF of viscoelastic fluid in a long channel.

We consider the motion of incompressible viscoelastic fluid containing ions K^+^ and Cl^−^ in a long channel of length *L*, height *H* and width *W* under externally applied potential difference *V*_0_ across the channel. We assume that the channel height is much smaller than both the length and the width (i.e., H≪L, H≪W), then the problem can be simplified to a 2D problem schematically shown in Figure 1. Cartesian coordinates O-*xy* are adopted with *y*-axis in the length direction and the origin fixed on one of the channel walls. 

The mass and momentum conservation equations for the fluid motion are

(1) ∇·u=0,

(2) $$\rho \left(\frac{\partial u}{\partial t}+u\cdot \nabla u\right)=-\nabla p+2\eta_{s}\nabla \cdot D+\nabla \cdot \tau +\rho_{e}E.$$

In the above, u and *p* are the velocity field and pressure, respectively; *ρ* denotes the fluid density; $\eta_{s}$ is the solvent dynamic viscosity; $D=\frac{1}{2}\left[\nabla u+{\left(\nabla u\right)}^{T}\right]$ denotes the deformation tensor; $\rho_{e}$ is the charge density within the electrolyte solution; E=−∇ϕ is the electric field with ϕ being the electric potential within the solution; τ is the extra polymeric stress tensor, which can be described by different constitutive models depending on the type of viscoelastic fluid such as Oldryod-B model, FENE-P model, PTT model and so forth. In general, τ can be written in terms of the conformation tensor c, a tensorial variable representing the macromolecular structure of the polymers. This study adopts the LPPT model to describe the viscoelastic fluid with

(3) $$\tau =\frac{\eta_{p}}{\lambda }\left(c-I\right),$$

where $\eta_{p}$ is the polymer dynamic viscosity and λ is the relaxation time. 

The evolution of the conformation tensor ***c*** for the LPTT model is governed by

(4) $$\frac{\partial c}{\partial t}+u\nabla \cdot c-\left(c\cdot \nabla u^{T}+\nabla u\cdot c\right)=-\frac{1}{\lambda }\left(1+\varepsilon \left(\mathrm{tr}\left(c\right)-3\right)\right)\left(c-I\right),$$

where ε is the extensibility parameter, and tr(***c***) is the trace of the conformation tensor ***c***. 

As shown in Figure 1, the solid surface in contact with a binary KCl electrolyte solution of bulk concentration $C_{0}$ will develop a layer with non-neutral charge density due to the electric interaction between the charged surface and the ions. This layer is referred to as electrical double layer (EDL). The electric potential ∅ within the electrolyte solution is governed by the Poisson equation:

(5) $$-\varepsilon_{f}\nabla^{2}\emptyset =F\left(z_{1}c_{1}+z_{2}c_{2}\right),$$

where $\varepsilon_{f}$ is the permittivity of the fluid, F is the Faraday constant, and $c_{1}$($c_{2}$) and $z_{1}\left(z_{2}\right)$ are the ionic concentration and the valence of K^+^ (Cl^−^) ions, respectively. 

The distribution of the ionic concentration is governed by the Nernst-Planck equation,

(6) $$\frac{\partial c_{i}}{\partial t}+\nabla \cdot \left(uc_{i}-D_{i}\nabla c_{i}-z_{i}\frac{D_{i}}{RT}Fc_{i}\nabla \emptyset \right)=0,\;\;i=1,2,$$

where *R* and *T* are, respectively, the gas constant and the absolute temperature; and $D_{i}$ is the diffusivity of the *i*th ionic species. 

The set of governing equations can be normalized by selecting $C_{0}$ as ionic concentration scale, RT/F as electric potential scale, the channel height H as length scale, $U_{0}=\varepsilon_{f}R^{2}T^{2}/\left(\eta_{0}HF^{2}\right)$ as the velocity scale, $\eta_{0}=\eta_{s}+\eta_{p}$ is the zero-shear rate total viscosity, and $\rho U_{0}{}^{2}$ as pressure scale. Then the dimensionless form of the governing Equations (1), (2) and (4)–(6) under steady state is obtained as

(7) $$\nabla^{\prime }\cdot u^{\prime }=0,$$

(8) $$u^{\prime }\cdot \nabla^{\prime }u^{\prime }=-\nabla^{\prime }p^{\prime }+\frac{\beta }{Re}{\nabla^{\prime }}^{2}u^{\prime }+\frac{\left(1-\beta \right)}{Re\cdot Wi}\nabla^{\prime }\cdot c-\frac{{\left(kH\right)}^{2}}{2Re}\left(z_{1}c_{1}^{\prime }+z_{2}c_{2}^{\prime }\right)\nabla {}^{\prime }\emptyset {}^{\prime },$$

(9) $$u{}^{\prime }\cdot \nabla {}^{\prime }c-\left(c\cdot \nabla {}^{\prime }u^{\prime }{}^{T}+\nabla {}^{\prime }u{}^{\prime }\cdot c\right)=-\frac{1}{Wi}\left(1+\varepsilon \left(\mathrm{tr}\left(c\right)-3\right)\right)\left(c-I\right),$$

(10) $${\nabla^{\prime }}^{2}\emptyset^{\prime }=\frac{{\left(kH\right)}^{2}}{2}\left(z_{1}c_{1}^{\prime }+z_{2}c_{2}^{\prime }\right),$$

(11) $$\nabla^{\prime }\cdot \left(u^{\prime }c_{i}^{\prime }-D_{i}^{\prime }\nabla^{\prime }c_{i}^{\prime }-z_{i}D_{i}^{\prime }c_{i}^{\prime }\nabla^{\prime }\emptyset^{\prime }\right)=0,\;\;i=1,2.$$

In the above, $u^{\prime }$ and *p*’, and $\emptyset {}^{\prime }$, and $c_{i}^{\prime }$ are the dimensionless velocity, pressure, electric potential, and the ionic concentration, respectively, and $D_{i}^{\prime }=D_{i}\eta_{0}F^{2}/\varepsilon_{f}R^{2}T^{2}$. The Debye length is $k^{-1}=\sqrt{\varepsilon_{f}RT/\sum_{i-1}^{2}F^{2}z_{i}^{2}C_{0}}$. The parameter β is the ratio of the solvent viscosity $\eta_{s}$ to the total viscosity $\eta_{0}$, i.e., $\beta =\frac{\eta_{s}}{\eta_{0}}$. The dimensionless parameters are Reynolds number $Re=\rho U_{0}H/\eta_{0}$, and Weissenberg number $Wi=\lambda U_{0}/H$. 

The boundary conditions are given as following.

On the charged wall,

(12) $$u{}^{\prime }=0,\;n\cdot \nabla^{\prime }p^{\prime }=0,\;n\cdot \nabla^{\prime }\emptyset^{\prime }=\sigma_{0}\cdot \frac{HF}{RT},n\cdot \nabla^{\prime }c_{i}^{\prime }=-z_{i}c_{i}^{\prime }n\cdot \nabla^{\prime }\emptyset^{\prime },\;n\cdot \nabla^{\prime }c=0,$$

where $\sigma_{0}$ is the surface charge density of the channel wall.

At the Anode (or Cathode),

(13) $$n\cdot \nabla^{\prime }u{}^{\prime }=0,\;\;p{}^{\prime }=0,\;\emptyset^{\prime }=V_{0}\cdot \frac{F}{RT}\;\left(\mathrm{or}\;0\right),c_{i}^{\prime }=1,\;n\cdot \nabla^{\prime }c=0.$$

where $V_{0}$ is the external electric potential applied at the anode. 

As the problem is symmetric, at the centerline of channel x=H/2, zero gradient is imposed on all variables. 

## 3. Numerical Method and Validation

The above coupled Equations (7)–(11) are solved by a new solver implemented in an open source CFD software OpenFOAM (FOAM-Extend 3.2, https://openfoam.org). For numerical simulation of viscoelastic fluid flow, the so-called high Weissenberg number problem (HWNP), due to the hyperbolic nature of the additional equations, the loss of symmetric positive definite (SPD) property and the unfaithful evaluation of conformation tensor ***c***, significantly impedes its accuracy and stability at high *Wi* [38]. A huge amount of effort has been made to resolve this problem when calculating the evolution of polymeric elastic stress, e.g., by introducing the artificial diffusion term [39,40], reconstructing better discretization schemes [41], and decomposing or reformulating the conformation tensor ***c*** [41,42]. In this study, log conformation reformulation (LCR) method [42] is implemented into the new solver. This method calculates the conformation tensor ***c*** by solving its logarithm instead of solving it directly, thereby, guaranteeing its SPD property automatically. Meanwhile, the deviation between polynomial fitting and exponential variation profiles of the conformation tensor ***c*** is eliminated. 

As the conformation tensor ***c*** is a SPD matrix, it can be decomposed as

(14) $$c=R\Lambda R^{T},$$

where ***R*** is an orthogonal matrix composed by the eigenvectors of ***c***, and Λ is a diagonal matrix whose diagonal elements are the eigenvalues of ***c***. The matrix logarithm of the conformation tensor ***c*** is introduced as

(15) $$\Psi =\log\left(c\right)=R\log\left(\Lambda \right)R^{T}.$$

Then, the evolution Equation (9) for the conformation tensor ***c*** can be reformulated in terms of this new variable Ψ as

(16) $$u^{\prime }\cdot \nabla^{\prime }\Psi -\left(\Omega \cdot \Psi -\Psi \cdot \Omega \right)-2B=-\frac{1}{Wi}e^{-\Psi }\left(1+\varepsilon \left(\mathrm{tr}\left(e^{\;\Psi }\right)-3\right)\right)\left(e^{\;\Psi }-I\right),$$

where Ω and B are the anti-symmetric matrix and the symmetric traceless matrix of the decomposition of the velocity gradient tensor $\nabla u^{\prime }$. The details of deriving Equation (16) and calculating Ω and B can be found in Fattal and Kupferman [42] and Zhang et al. [43]. After Ψ is solved, the conformation tensor **c** can be recovered from matrix-exponential of Ψ as

(17) c=exp(Ψ).

To improve the convergence and the stability of the calculation, the convection terms in Equations (8), (11) and (16) are discretized by QUICK [44], Gauss linear, and MINMOD scheme [45], respectively, while the diffusion terms are discretized by Gauss linear scheme. The coupling of velocity and pressure fields is solved by PISO algorithm [46,47]. Orthogonal mesh is used with much denser mesh distributed near the charged wall. 

**Figure 2:**
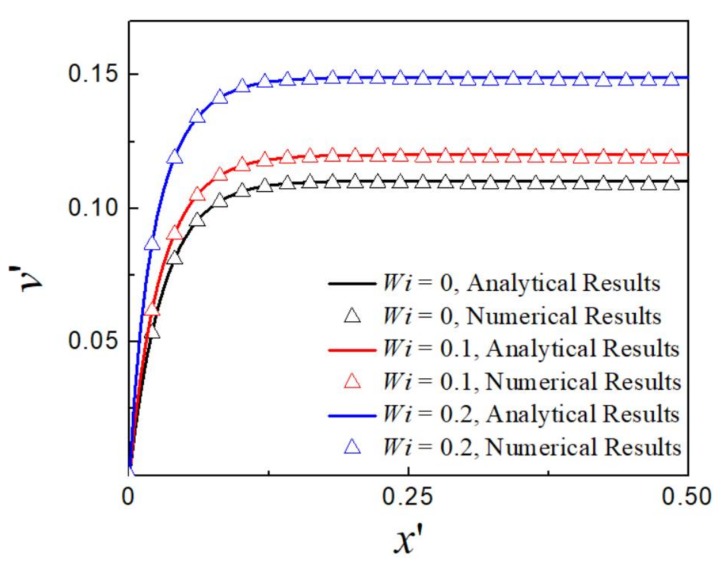
Dimensionless *y*-component velocity profile for Newtonian and viscoelastic fluids at different *Wi*: analytical results of Afonso et al. [31], (solid line) and current numerical results (symbol).

To check the validity of the developed code, we first compare the numerical predictions with the analytical results of Afonso et al. [31], who derived analytical solution for EOF with the simplified PTT (sPTT) model in a two-dimensional microchannel with assumptions of low zeta potential and thin EDL so that the Poisson-Nernst-Planck equations can be simplified to Poisson-Boltzman equation. In the current simulation, the geometry of the channel is set as height H= 100 nm and length *L* = 300 nm. For comparison with the sPTT model in the reference, the solvent viscosity is set to 0, i.e., β=0. Other parameters are set as $D_{1}=1.96\times 10^{-9}{\;m}^{2}\cdot s^{-1},\;D_{2}=2.03\times 10^{-9}{\;m}^{2}\cdot s^{-1}$, T=300 K, $F=96,485\;C\cdot {\mathrm{mol}}^{-1}$, $\varepsilon_{f}=7.08\times 10^{-10}{\;\mathrm{CV}}^{-1}\cdot m^{-1}$. The electric potential at inlet is set to 0.05 V and the outlet is grounded. The zeta potential is set to −4.36 mV on the wall. Figure 2 shows the predicted dimensionless *y*-component velocity distribution in the middle of the channel at kH/2=16.45 (kH/2 is ratio of the half of the channel height to EDL thickness) in comparison with the corresponding analytical solution for Newtonian fluid (*Wi* = 0) and viscoelastic fluid at ε=1  and various *Wi*. As the EDL is relatively thin, the velocity profile is plug-like, and increases with higher *Wi*. It is clearly seen that our numerical results agree well with analytical solutions of Afonso et al. [31] for both Newtonian and viscoelastic fluids at different *Wi*. 

## 4. Results and Discussion

The validated solver is then applied to investigate the effects of *Wi*, the extensibility parameter ε, and the viscosity ratio β on the EOF of viscoelastic fluid in a long nanoslit with the consideration of EDL overlap. For illustration, a channel of length *L* = 5 μm and height *H* = 100 nm is considered, which is long enough to eliminate the end effect. The fully developed EOF for different *Wi* is simulated under different EDL conditions: *C*_0_ = 0.01, 0.1, and 10 mM, corresponding to kH/2=0.52, 1.64, and 16.45. Other parameters are set as $\sigma_{0}=-0.01\;C/m^{2},$ $V_{0}=0.05\;V$, ε=0.25 and β=0.1 unless they are specifically stated.

**Figure 3:**
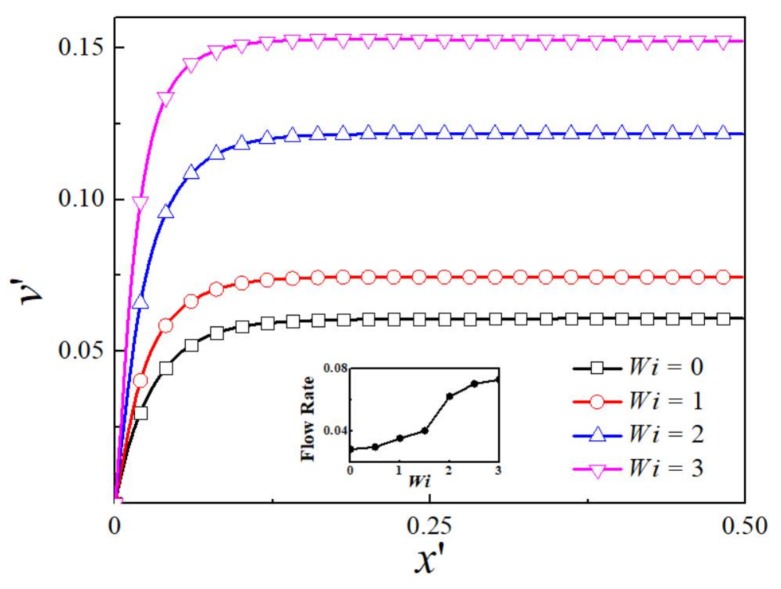
The distribution of dimensionless *y*-component velocity for various *Wi* at kH/2=16.45. *Inset*: Dependence of dimensionless flow rate on *Wi*.

Figure 3 shows the dimensionless *y*-component velocity profile at different *Wi* at kH/2=16.45. It is observed that the velocity increases sharply near the wall and reaches a plateau value, revealing a plug-like profile. This is because the EDL thickness is much smaller than the channel height, so the electric charge is neutral within the channel outside the EDL region. The plateau value increases with an increase in Weissenberg number. The maximum velocity at the centerline for viscoelastic fluid at *Wi* = 3 is 2.50 times of that for Newtonian fluid. The variation of flow rate with *Wi* is shown in inset graph of Figure 3. As *Wi* increases, the flow rate demonstrates a monotonous growth over the whole range of *Wi* investigated. 

**Figure 4:**
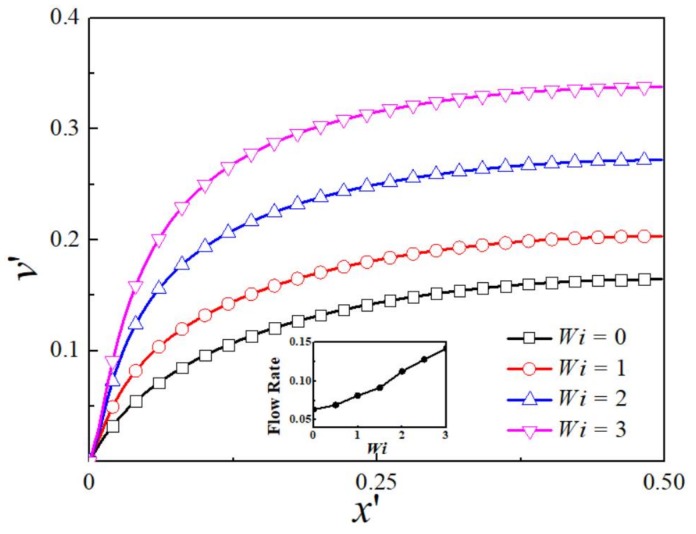
The distribution of dimensionless *y*-component velocity for various *Wi* at kH/2=1.64. *Inset*: Dependence of dimensionless flow rate on *Wi*.

Figure 4 depicts the dimensionless *y*-component velocity profile for different *Wi* at kH/2=1.64. When the EDL thickness increases to the level comparable to the channel height, the plug-like velocity changes to the parabolic-like velocity profile. For all values of *Wi*, the velocity keeps increasing from the wall to the channel center. As the EDL is almost overlapping under this condition, the whole channel is filled with more counter-ions, so the velocity is not uniform even near the channel centerline. The maximum velocity at the channel center for viscoelastic fluid at *Wi* = 3 is 2.05 times of that for Newtonian fluid. The flow rate also monotonously increases with *Wi* as shown in inset graph of Figure 4. Compared to the results of thin EDL thickness, the flow rate is much higher due to the increase of counter-ions within the whole channel. 

**Figure 5:**
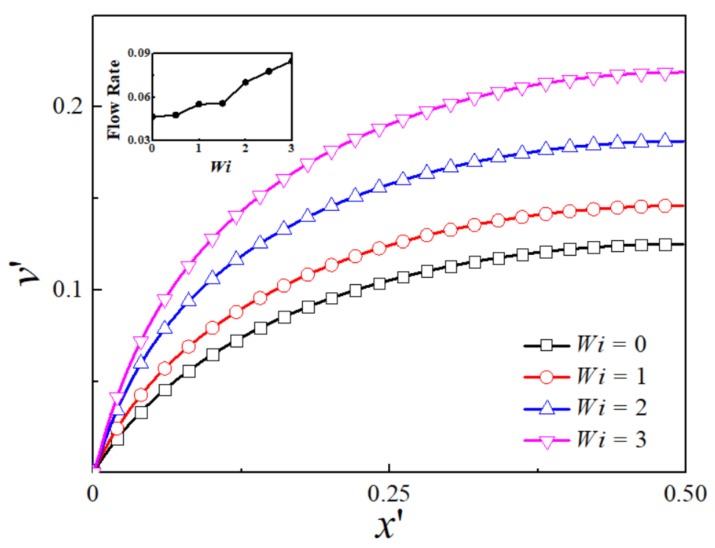
The distribution of dimensionless *y*-component velocity for various *Wi* at kH/2=0.52. *Inset*: Dependence of dimensionless flow rate on *Wi*.

This trend is more obvious for the condition with apparent EDL overlap when more counter-ions are accumulated within the channel [20], as can be seen in Figure 5 for kH/2= 0.52 where the EDL is highly overlapped. The velocity also increases slowly across the whole channel, resembling that of the pressure-driven flow. The maximum velocity at the centerline for viscoelastic fluid at *Wi* = 3 is 1.75 times of that for Newtonian fluid. The inset figure depicts the dependence of flow rate on *Wi*. Table 1 summarizes the maximum velocity at the centerline and the enhancement of the maximum velocity for the three cases with different EDL thickness when *Wi* = 3. Comparing the three cases, the enhancement of maximum velocity at the centerline for viscoelastic fluid decreases as the EDL thickness increases. This is because the velocity is increasing slowly across the whole channel instead of increasing sharply near the wall as EDL becomes overlapped, thus the overall shear rate is smaller, especially near the wall. As the mechanism of the velocity increase is due to the shear thinning effect, it is expected that the viscoelasticity has larger effect on the case that has larger shear rate. 

**Figure 6:**
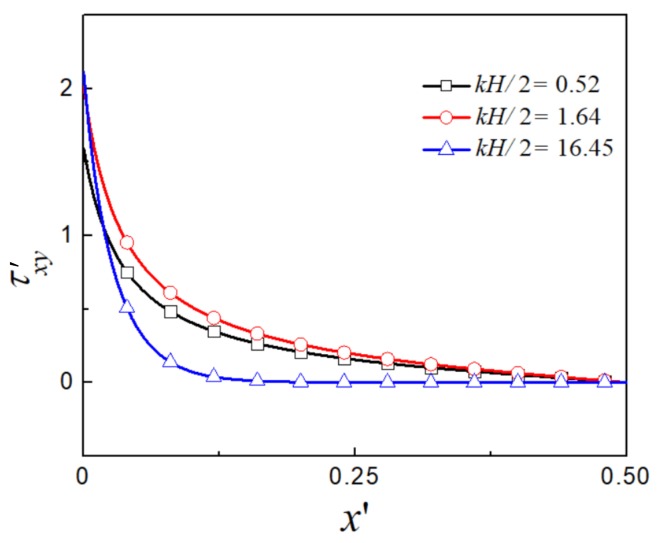
The distribution of the total dimensionless shear stress for different values of kH/2.

The distribution of the total dimensionless shear stress for different values of kH/2 is shown in Figure 6. The shear stress is independent of the rheological parameter, while is affected by the EDL thickness. For thin EDL, i.e., kH/2=16.45, the shear stress is zero almost within the entire channel and increases sharply near the wall. When EDL thickness is comparable to the channel height, the shear stress increases from 0 from the centerline to the wall across the entire channel. This is because the electric body force in the latter case is distributed in the entire channel compared to the thin EDL case, where the electric body force is only accumulated near the channel wall. At the wall, the dimensionless shear stress increases as the EDL thickness decreases, and the shear stress is suppressed within the EDL region near the wall. 

After analyzing the velocity profile and the shear stress, the shear viscosity is calculated from

(18) $$\eta =\frac{\tau_{xy}^{\prime }}{dv{}^{\prime }/dx{}^{\prime }}.$$

**Figure 7:**
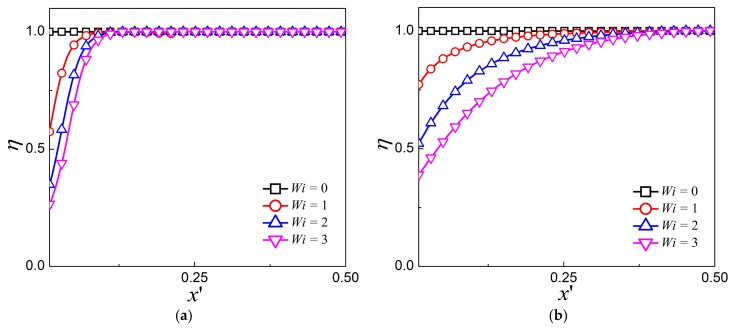
The shear viscosity profile for various *Wi* at (**a**) kH/2=16.45 and (**b**) kH/2=0.52.

Figure 7 depicts the variation of shear viscosity for various *Wi* under different values of kH/2. The results clearly illustrate that the shear viscosity remains unit one within the whole channel for Newtonian fluid. For viscoelastic fluid, the shear viscosity remains unit one at the centerline, and decreases monotonically from the centerline to the wall, where the shear rate is larger. As *Wi* increases, a more apparent decrease is observed. This is the shear thinning characteristics of the viscoelastic fluid, leading to the increase of the velocity. Comparing the variation of shear viscosity η for different EDL thickness, it can also be noticed that η decreases rapidly near the wall for thin EDL and decreases gradually within the entire channel for larger EDL thickness. 

**Figure 8:**
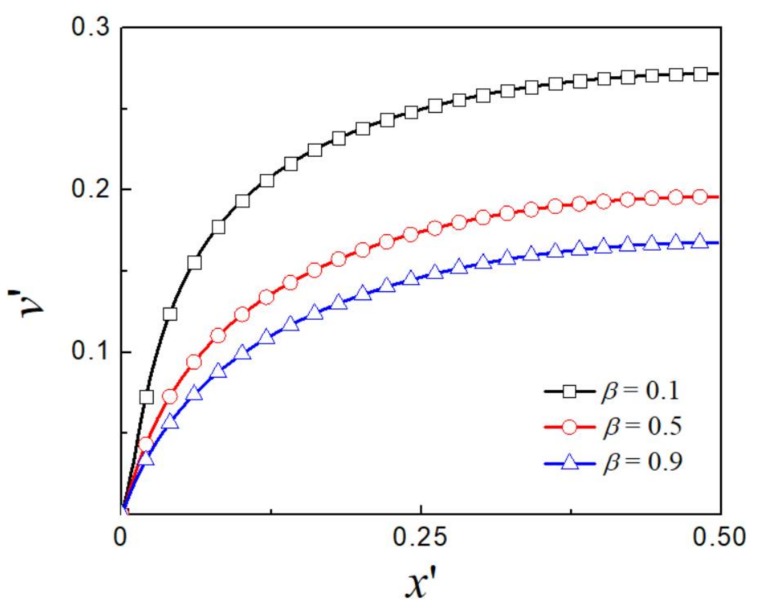
The distribution of dimensionless *y*-component velocity for various β at kH/2=1.64 and ε=0.25.

**Figure 9:**
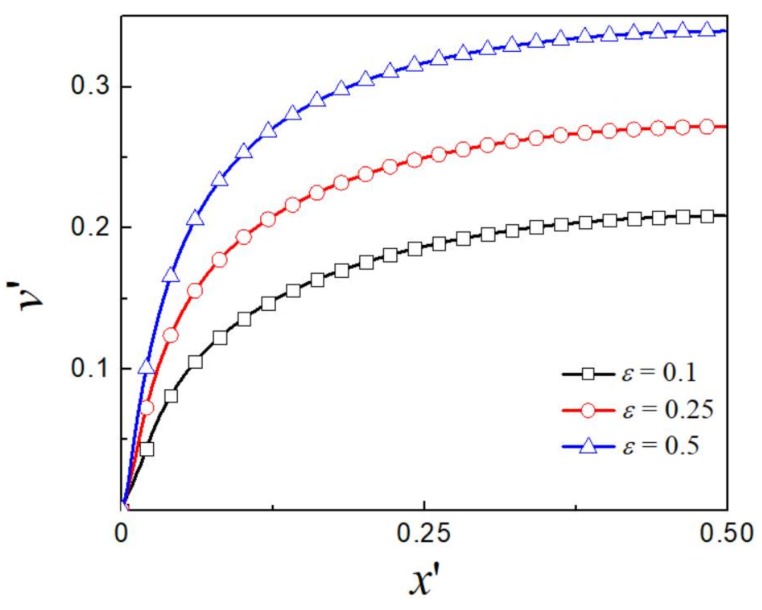
The distribution of dimensionless *y*-component velocity for various ε at kH/2=1.64 and β=0.1.

The EOF of viscoelastic fluid is dependent on the rheological parameter of the fluid. Figure 8 and Figure 9 present the dimensionless velocity profiles for various values of viscosity ratio β and extensibility parameter ε while keeping *Wi* = 2 and the EDL thickness unchanged at kH/2=1.64. Significant flow enhancement is seen as β decreases and ε increases. The limiting case of β=1 or ε=0 is corresponding to the Newtonian fluid or viscoelastic fluid without shear thinning behavior.

## 5. Conclusions

Numerical study for the EOF of viscoelastic fluid in a long nanochannel is conducted to investigate the effects of rheological properties of LPTT fluid on the fully developed EOF. The non-linear Poisson-Nernst-Planck (PNP) equations are adopted to describe the electric potential and ionic concentration distribution within the channel without using the assumptions of low surface (or zeta) potential and thin EDL. EDL overlapping is considered in this study due to the use of the PNP equations. When the EDL is not overlapped, the velocity profiles for both Newtonian and viscoelastic fluid of different Weissenberg number are plug-like with a rapid increase within the EDL. Apparent increase of velocity is observed for viscoelastic fluid compared to the Newtonian fluid, and this is due to the shear thinning effect. The increase of maximum velocity at the center of the channel is less significant for thicker EDL. EOF velocity increases with an increase in the polymer extensibility (ε) and a decrease in the viscosity ratio (β). Since the straight channel has a uniform cross-section and the flow is steady-state, the elastic effect on EOF is not demonstrated in the current study. 

## Figures and Tables

**Table 1:** The maximum velocity at the centerline and the enhancement of the maximum velocity for different kH/2 when *Wi* = 3.

Variable	kH/2=16.45	kH/2=1.64	kH/2=0.52
Maximum velocity	0.15	0.34	0.22
Enhancement of the maximum velocity	2.50	2.05	1.75
